# Demographic and Genetic Predictors of ADHD Diagnoses and Medications in Electronic Health Records

**DOI:** 10.21203/rs.3.rs-7520845/v1

**Published:** 2025-10-30

**Authors:** Olivia R. Nicastro, Makayla Reed, Zeal Jinwala, Penn Medicine BioBank, Regeneron Genetics Center, Rachel L. Kember, Emily E. Hartwell

**Affiliations:** University of Pennsylvania; University of Pennsylvania; University of Pennsylvania; University of Pennsylvania; University of Pennsylvania; Philadelphia VA Medical Center

**Keywords:** Attention-deficit/hyperactivity disorder, prescription stimulants, polygenic scores, prescription misuse

## Abstract

**Background:**

The rise in stimulant prescriptions, which are commonly prescribed for attention-deficit/hyperactivity disorder (ADHD), has raised concerns due to their high potential for abuse.

**Methods:**

This study utilized data from the Penn Medicine BioBank (PMBB) to investigate prescription stimulant use by identifying: (1) individuals with an International Classification of Diseases (ICD) code for ADHD, (2) individuals prescribed a stimulant, (3) individuals with both an ICD code for ADHD and a stimulant prescription, (4) individuals without an ICD code for ADHD but with a stimulant prescription, and (5) a control group. We assessed differences in demographics, medication type, and dosages amongst these groups. For participants with available genetic data, we calculated polygenic scores (PGS) for ADHD and used regression models to examine whether PGS for ADHD is associated with ADHD diagnosis and stimulant medication use.

**Results:**

Demographic differences between the five groups were identified. Individuals with an ADHD diagnosis were more likely to be younger, female, and White, compared to the control group. Individuals with ICD codes for ADHD were prescribed significantly higher average doses of stimulants compared to those without ADHD. PGS for ADHD was significantly associated with the likelihood of receiving an ADHD diagnosis, using a stimulant, using a stimulant in the absence of an ADHD diagnosis, and receiving a lower dosage of a stimulant.

**Conclusions:**

Findings from this research will contribute to discussions surrounding the role of genetic predisposition and demographic disparities in ADHD diagnosis and treatment.

## Background

Changes in mental health awareness, diagnostic practices, and treatment strategies have coincided with a notable increase in the use of medications for psychiatric conditions. Stimulant prescriptions, for example, have grown by 60% from 2012 to 2023 in the United States (U.S. Department of Justice, 2023), and the percentage of medical visits in which stimulants were prescribed has increased from 0.11% (1994–1997) to 0.70% (2006–2009) (Olfson et al., 2013). Common stimulants include methylphenidate (e.g., Ritalin) and amphetamine compounds (e.g., dextroamphetamine; Adderall), both of which have a high potential for abuse and physiological dependence (Weyandt et al., 2016). Increases in stimulant prescriptions from 2021 to 2022 were noted for dexmethylphenidate (11.5%), methylphenidate HCl (9.1%), and amphetamine/dextroamphetamine (8.5%). The most dispensed stimulants for ADHD treatment in 2022 were amphetamine/dextroamphetamine (U.S. Department of Justice, 2023).

Research has also found that across all stimulant products, there was an initial increase in average daily dose from 2012 to 2013, followed by a consistent overall 8.7% decrease in AvDD from 2013 to 2022. The most notable decreases occurred from 2017 to 2018 and 2020 to 2022 (U.S. Department of Justice, 2023).

Historically, the highest rates of stimulant prescriptions were observed in individuals aged 5–19 and 15–24, respectively, regardless of sex. While rates remained stable or decreased for younger age groups (≤ 24 years) between 2016 and 2020, modest increases were seen among older adults (25–64 years). However, from 2020 to 2021, there was a significant rise in stimulant prescriptions among females aged 15–44 and 50–54, and among males aged 25–44 and 50–54 (Danielson et al., 2023). The greatest increase in the number of individuals receiving prescriptions for stimulants occurred among women aged 35–64 years between 2019 and 2022 (Han et al., 2025). Additionally, American Indian or Alaskan Native, Asian, and White individuals all experienced an increase in prescriptions across various age groups, but only White individuals showed a significant rise among those aged 65 and older (Brumbaugh et al., 2022). Prior research has also found that U.S. states with larger Hispanic populations had lower stimulant use (Piper et al., 2018).

Stimulants are routinely used to treat attention-deficit/hyperactivity disorder (ADHD), a common childhood disorder that often persists through adolescence and adulthood (Stevens et al., 2013). The criteria for an ADHD diagnosis include: (1) at least six symptoms of inattention and/or hyperactivity-impulsivity for children aged 16 and younger, or (2) at least five symptoms for adolescents aged 17 and older and adults (Diagnosing ADHD, 2024). The surge in stimulant prescriptions might suggest a corresponding rise in ADHD diagnoses, however, studies indicate that while stimulant prescriptions in the United States increased by 250% between 2006 and 2016, ADHD diagnoses saw only a minimal uptick during the same period (Brumbaugh et al., 2022).

Both genetic and environmental factors contribute to the development of ADHD, with an estimated heritability of 74% (Faraone & Larsson, 2019). Genome-wide association studies (GWAS) of ADHD have identified 27 risk loci for the disorder (N = 38,691; Demontis et al., 2023). These studies also show that about a third of ADHD’s heritability is due to a polygenic component comprising many common variants each having small effects (Faraone & Larsson, 2019). However, the extent to which genetic factors contribute to the use or dosage of stimulant medication is unknown.

This study uses data from the Penn Medicine BioBank (PMBB) to assess differences in age, race, ethnicity, sex, medication type, and medication dosage amongst (1) individuals with an International Classification of Diseases (ICD) code for ADHD, (2) individuals prescribed a stimulant, (3) individuals with both an ICD code for ADHD and a stimulant prescription, (4) individuals without an ICD code for ADHD but with a stimulant prescription, and (5) a control group. We further assess whether individuals with a higher genetic liability for ADHD (as denoted by a polygenic score [PGS]) have a higher likelihood of receiving an ADHD diagnosis, stimulant medication, a stimulant prescription without an ADHD diagnosis, and higher stimulant dosages.

## Methods

### Participants.

The current study utilized the demographically diverse PMBB (N = 44,255), which is an ongoing electronic health record biobank created in 2013 at the University of Pennsylvania. The PMBB integrates a wide array of health-related data, including diagnosis codes, laboratory results, prescribing records, imaging studies, and lifestyle factors, alongside genomic and biomarker information (Verma et al., 2022). All participants provide informed consent to being in the biobank at time of enrollment and provided a blood or tissue sample as well as permission to access their electronic health record (EHR). This research was conducted in accordance with the Declaration of Helsinki and was approved by the University of Pennsylvania’s Institutional Review Board.

### Phenotypes.

We selected individuals with ADHD diagnoses, as well as those with other conditions for which stimulant medications are prescribed, based on the ICD codes listed in Supplementary Table 1. ICD codes are a standardized system for classifying and coding medical conditions (International Classification of Diseases (ICD), n.d.). Individuals with 1 or more ICD codes listed were defined as a case for ADHD. To identify individuals prescribed stimulants, we examined prescription records for participants who received any medications from the stimulant groups listed in Supplementary Table 2. Individuals were defined as receiving a stimulant medication if they received 1 or more of these medications. We also extracted information about medication type, length of prescription, and dosage. We then standardized all daily dosages to an Adderall-equivalent dose (ADHD Medication Calculator, n.d.).

### Genotyping and Quality Control.

DNA was extracted from blood and samples were genotyped using the Illumina Global Screening Array (GSA V2) at the Regeneron Genetics Center (RGC). Samples were removed if the genotyping call rate was < 90% or if there were sex discrepancies, and SNPs were removed if the marker call rate < 95%. Genotypes were phased (using EAGLE) and imputed to the TOPMed Reference Panel (Freeze 5) on the TOPMed Imputation Server. One individual from each pair of related individuals (pi-hat threshold of 0.25) was removed from the analysis. PCA was conducted using smartpca and the Hapmap3 dataset to determine genetic ancestry.

### Polygenic Scores.

A PGS is a single number that estimates a person’s inherited risk for a condition based on the combined effect of many small genetic differences across their DNA. In order to calculate PGS, GWAS summary statistics for ADHD were obtained from Demontis et al. (2023; cases = 38,691, controls = 186,843). Because GWAS summary statistics are available for European (EUR) ancestry individuals only, we only utilized EUR individuals from the PMBB (N = 29,355) for the genetic analyses. We used PLINK v.190 to calculate ancestry-specific principal components (PCs) to use as covariates. Polygenic scores were calculated using PRS-CS (Ge et al., 2019) with EUR GWAS summary statistics and the 1000G EUR LD reference panel. Phi for all traits was fixed to 1e-2, with default thresholds used for everything else. We retained SNPs present in the Hapmap reference panel (n = 1,120,629).

### Statistical Analyses.

From the total PMBB sample, we identified five groups: (1) individuals with an ICD code for ADHD, (2) individuals prescribed a stimulant, (3) individuals with both an ICD code for ADHD and a stimulant prescription, (4) individuals without an ICD code for ADHD but with a stimulant prescription, and (5) the control group which comprised of remaining individuals who did not meet any of the above criteria (Table 1). For individuals prescribed a stimulant, we removed individuals receiving oral solutions and individuals with prescription quantities of 0 or > 400. For individuals receiving between 30–360 pills, we assumed a 30-day prescription to calculate their daily dose. For individuals receiving between one and 29 pills, we assumed one pill per day for their daily dose. This study was not preregistered.

**Figure 1:**
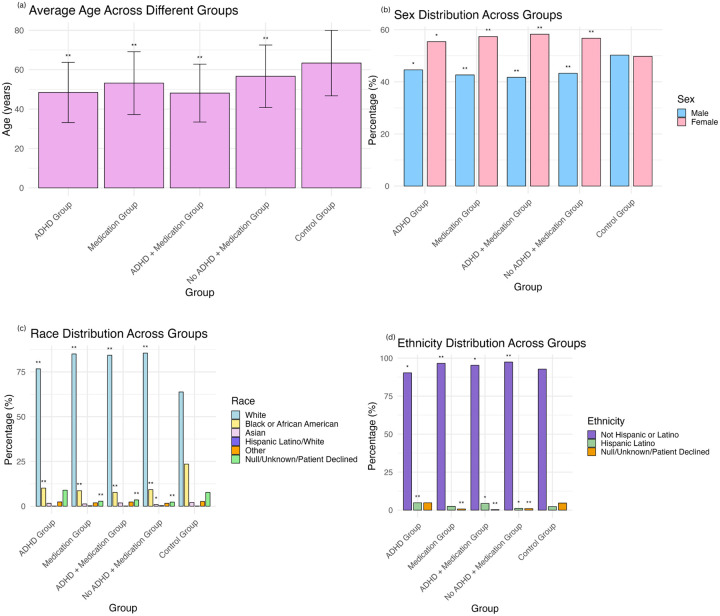
Demographics by Group Demographic characteristics of the (1) ADHD group, (2) Medication group, (3) ADHD + Medication group, (4) No ADHD + Medication group, and (5) Control group. Statistical significance designations refer to comparisons to the control group. Panels display: (a) average age, (b) sex distribution, (c) race distribution, and (d) ethnicity distribution across groups. * p < 0.05 ** p < 0.001

Table 1 and Fig. 1 present demographic characteristics of the study sample. We conducted two-sample t-tests to assess age differences between the groups and chi-square tests to explore categorical variables across the groups. Additionally, we used t-tests to assess differences in the average stimulant dosage between participants with and without ICD codes for ADHD.

For the EUR individuals in PMBB, we examined whether PGS for ADHD was associated with having an ADHD diagnosis, stimulant medication use, and a greater likelihood of receiving a stimulant prescription without an ADHD diagnosis using logistic regression models in R with the glm() package. Lastly, we conducted an exploratory linear regression to examine the relationship between medication dosage and PGS. This analysis was conducted first across all individuals receiving stimulant medication, and then separately among only those diagnosed with ADHD. All analyses were conducted in RStudio (Version 2023.6.2.561).

## Results

### Sample

Significant age differences were found across all groups compared to controls. Specifically, individuals in all groups were significantly younger than the control group: (1) individuals with an ICD code for ADHD (*t*(909.37) = −28.41, *p* < .001), (2) individuals prescribed a stimulant (*t*(1562.90) = −24.08, *p* < .001), (3) individuals with both an ICD code for ADHD and a stimulant prescription (*t*(612.24) = −25.12, *p* < .001), and (4) individuals without an ICD code for ADHD but with a stimulant prescription (*t*(899.79) = −12.43, *p* < .001). Additionally, (3) individuals with both an ICD code for ADHD and a stimulant prescription were significantly younger than (4) individuals without an ICD code for ADHD but with a stimulant prescription (*t*(1330.10) = −10.52, *p* < .001) (Table 1, Fig. 1).

We also identified significant differences across groups for race and ethnicity. Individuals with an ADHD diagnosis or individuals receiving a stimulant prescription were more likely to be White (ADHD: *X*^*2*^ = 61.17, *df* = 1, *p* < .001; stimulant: *X*^*2*^ = 275.16, *df* = 1, *p* < .001) and less likely to be Black (ADHD: *X*^*2*^ = 84.66, *df* = 1, *p* < .001; stimulant: *X*^*2*^ = 174.27, *df* = 1, *p* < .001) (Table 1, Fig. 1). Further analysis revealed that individuals with an ADHD diagnosis were more likely to be Hispanic or Latino (*X*^*2*^ = 21.10, *df* = 1, *p* < .001), while individuals receiving a stimulant prescription were more likely to not be Hispanic or Latino (*X*^*2*^ = 30.20, *df* = 1, *p* < .001) (Table 1, Fig. 1). Additionally, individuals with an ADHD diagnosis or individuals receiving a stimulant prescription were more likely to be female (ADHD: *X*^*2*^ = 10.58, *df* = 1, *p* = .001; stimulant: *X*^*2*^ = 32, *df* = 1, *p* < .001) (Table 1, Fig. 1).

### Medications and Dosage

We analyzed differences in stimulant prescriptions among individuals with and without an ADHD diagnosis. The only difference in the type of medication prescribed between these two groups was that a single individual without an associated ICD code for ADHD received methamphetamine hydrochloride, whereas no one with an ADHD diagnosis received this medication. Notably, this individual was not prescribed any other stimulant medications and did not have any other diagnoses that would constitute a stimulant prescription.

We further investigated whether individuals prescribed stimulants without an ADHD diagnosis had other conditions that might justify stimulant use. Among the 862 individuals in this group, 381 (44.2%) had ICD codes for alternative disorders such as narcolepsy, obesity, or treatment-resistant depression, which could potentially warrant stimulant prescriptions (Table 2). In comparison, a similar proportion of individuals with an ADHD diagnosis (50.98%) had these alternative diagnoses (Table 2). Therefore, 55.8% of individuals without an ADHD diagnosis did not have an alternative diagnosis that would explain the stimulant use. Lastly, individuals with ICD codes for ADHD were prescribed significantly higher average doses of stimulants (M = 8.51) compared to those without ADHD (M = 6.80; *t*(941.18) = 3.60, p < .001).

### Genetic Analyses

**Figure 2:**
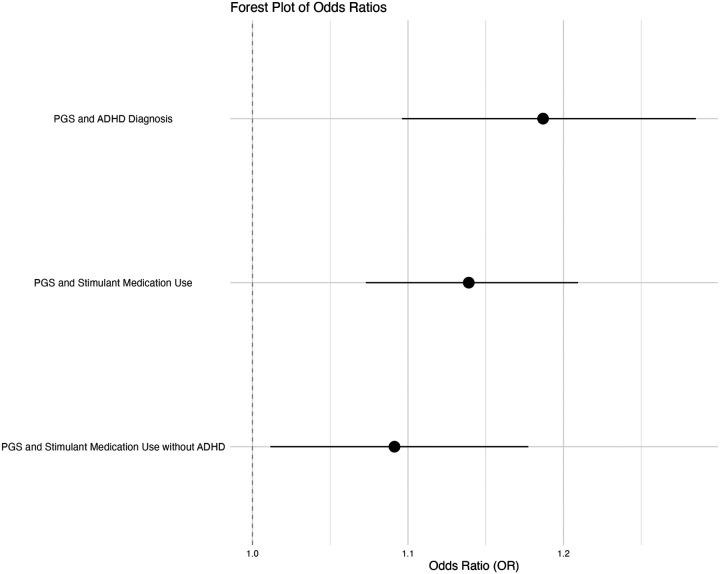
Forest Plot of Odds Ratios Note. Logistic regressions between PGS for ADHD and the (1) likelihood of receiving an ADHD diagnosis, (2) using a stimulant, and (3) using a stimulant without an ADHD diagnosis.

In the EUR ancestry individuals, PGS for ADHD was significantly positively associated with the likelihood of receiving an ADHD diagnosis (OR = 1.19, 95% *CI* [1.10, 1.29], *p* < .001) and the likelihood of receiving a stimulant prescription (OR = 1.14, 95% *CI* [1.07, 1.21], *p* < .001) (Fig. 2). Furthermore, stimulant use even in the absence of an ADHD diagnosis was positively associated with PGS for ADHD (OR = 1.09, 95% *CI* [1.01, 1.18], *p* = 0.024) (Table 3, Fig. 2). A significant association between PGS and stimulant medication dosage was identified. Specifically, as PGS for ADHD increases, stimulant dosage decreases (β = − 0.22, *p* = 0.014, 95% CI [–0.40, − 0.05]). This finding suggests that individuals with lower genetic liability for ADHD received higher Adderall adjusted AvDD of stimulant medications. Restringing this analysis to only individuals with an ADHD diagnosis that were prescribed a stimulant medication, the association between PGS and stimulant dosage was no longer statistically significant (β = − 0.22, *p* = 0.054, 95% CI [–0.004, 0.44]).

## Discussion

This study utilized 44,255 individuals from the PMBB to examine demographic and 29,355 genotyped individuals to assess genetic predictors of receiving a stimulant prescription with and without a diagnosis of ADHD. We observed significant differences for all demographic variables examined, suggesting that there may be demographic disparities in ADHD diagnosis and treatment. Furthermore, higher genetic liability for ADHD as captured by PGS was associated with an increased likelihood of ADHD diagnosis and stimulant prescription use, but a decreased dosage of those medications.

All examined groups were significantly younger than the control group, with the youngest group being individuals diagnosed with ADHD and using prescription stimulants. Our findings align with the pre-existing literature documenting a rise in stimulant prescriptions among females aged 15–44 and 50–54, and among males aged 25–44 and 50–54 (Danielson et al., 2023. The average age for individuals taking prescription stimulants was 53.17 which aligns with research showing a significant increase in stimulant prescriptions among adults aged 50–54 (Danielson et al., 2023). However, this finding may also reflect the older age distribution of individuals enrolled in the PMBB.

Individuals with an ADHD diagnosis or those prescribed a stimulant were more likely to be White and less likely to be Black. Prior research has indicated there are higher rates of stimulant prescriptions among White individuals, though additional work is needed to clarify patterns across other racial groups (Brumbaugh et al., 2022). Individuals diagnosed with ADHD were also more likely to identify as ethnically Hispanic or Latino. This finding contrasts with earlier studies that have linked Hispanic ethnicity to lower rates of ADHD diagnosis, highlighting the need for further exploration (Piper et al., 2018). In regard to sex, individuals with an ADHD diagnosis or those receiving stimulant prescriptions were more likely to be female than the control group. Given prior observations that females have historically been underdiagnosed relative to males (Martin, 2024), this result warrants closer examination. An increase in the number of females diagnosed or receiving treatment may be due to improved awareness of how ADHD presents in females.

Individuals in the PMBB without an ADHD diagnosis who received a stimulant did not have other conditions commonly treated with stimulants at higher rates than those diagnosed with ADHD. Thus, the presence of other diagnoses does not fully explain the stimulant use in this group. One possible explanation could be that these individuals have an ADHD diagnosis or another off-label indication that has not been captured in the EHR. However, individuals without ICD codes for ADHD were prescribed significantly lower average doses of stimulants compared to those with an ADHD diagnosis, indicative of differences in prescribing practices. Further research is therefore needed to explore why that pattern exists.

Our results showed that higher PGS for ADHD is associated with an increased likelihood of receiving an ADHD diagnosis, consistent with previous research (Faraone & Larsson, 2019). Higher PGS was associated with stimulant prescription use as well, which suggests that genetic predisposition, as indicated by higher PGS for ADHD, is predictive of both the likelihood of receiving an ADHD diagnosis and using stimulant medication.

While there is a growing body of research examining the off-label uses of stimulants, few studies have explored the potential genetic influence on the prescription of stimulants in individuals without a formal ADHD diagnosis (Panther et al, 2017). Our finding that individuals with higher PGS are more likely to use stimulant medications even without an ADHD diagnosis offers a novel contribution to the field, suggesting that genetic factors could contribute to stimulant prescriptions beyond formal ADHD diagnoses captured in the EHR. Genetic predisposition may play a role not only in the diagnosis of ADHD but also in the broader patterns of stimulant use, a finding that warrants further investigation into the factors driving stimulant prescriptions beyond diagnostic criteria.

Interestingly, higher PGS was associated with lower stimulant dosage, suggesting that individuals with lower genetic liability receive higher doses of stimulant medications. One possible explanation is that these individuals are more sensitive to stimulant effects, allowing lower doses to manage symptoms effectively. Alternatively, those with higher PGS may be identified and treated earlier in life, leading to more personalized and conservative dosing strategies over time.

This study’s strengths include the use of a large, diverse, longitudinal biobank that enabled robust statistical analyses which increased confidence in the results. Additionally, our investigation into medication differences between individuals with and without ADHD diagnoses provides novel insights into stimulant prescribing patterns. However, the genetic analyses were restricted to individuals of European ancestry, which limits the generalizability of our results across diverse populations. Furthermore, reliance on the EHR means that differences in clinician practices may have influenced observed disparities in diagnosis and treatment. We are also unable to capture diagnoses or pharmacy records occurring outside of Penn Medicine, which may cause omission or misclassification of key phenotypes.

## Conclusions

Overall, this study contributes to existing knowledge by highlighting the intersection of ADHD diagnosis and prescription stimulants. The results suggest that demographic variables, including PGS values for ADHD, are differentially associated with both the likelihood of receiving an ADHD diagnosis and the prescription of stimulant medications, including in individuals without a formal ADHD diagnosis. These findings underscore the need for further research into the factors influencing stimulant prescribing practices, particularly the role of genetic predisposition and demographic disparities in ADHD diagnosis and treatment.

## Supplementary Files

This is a list of supplementary files associated with this preprint. Click to download.

- SupplementaryTables.docx
- PMBBAuthorcontributions.docx

## Figures and Tables

**Table 1:** Demographic Differences by Group

Characteristic	ADHD Group (n = 868)	Medication Group (n = 1,454)	ADHD + Medication Group (n = 592)	No ADHD+ Medication Group (n = 862)	Control Group (n = 42,525)
Age (Mean ± SD)	48.45 +−15.27**	53.17 +−15.95**	48.11 +−14.70**	56.65+−15.86**	63.35 +−16.61
Sex					
Male (%)	44.59%*	42.64%**	41.72%**	43.27%**	50.22%
Female (%)	55.41%*	57.36%**	58.28%**	56.73%**	49.78%
Race					
White (%)	76.73%**	85.01%**	84.29%**	85.50%**	63.80%
Black or African American (%)	10.14%**	8.67%**	7.77%**	9.28%**	23.52%
Asian (%)	1.61%	1.31%	1.86%	0.93%*	2.14%
Hispanic Latino/White (%)	0.12%	0.28%	0.17%	0.35%	0.16%
Other (%)	2.42%	1.93%	2.37%	1.63%	2.66%
Null/Unknown/Patient Declined (%)	9.00%	2.82%**	3.54%**	2.32%**	7.74%
Ethnicity					
Not Hispanic or Latino (%)	90.32%*	96.56%**	95.27%*	97.45%**	92.76%
Hispanic Latino (%)	4.84%**	2.48%	4.39%*	1.16%*	2.36%
Null/Unknown/Patient Declined (%)	4.84%	0.76%**	0.34%**	1.04%**	4.67%

Note. Statistical significance designations refer to comparisons to the control group.

* *p* < 0.05

** *p* < 0.001

**Table 2:** Other Diagnoses Constituting a Stimulant Prescription by Group

Disorder	ADHD Group	Medication Group	ADHD + Medication Group	No ADHD + Medication Group	Control Group
**Binge Eating Disorder**	0.58%	0.75%	0.84%	0.93%	0.14%
**Bipolar Disorder**	14.63%	10.52%	13.34%	6.38%	2.25%
**Chronic Fatigue Syndrome**	5.18%	6.53%	5.24%	7.89%	2.12%
**Narcolepsy**	7.95%	8.15%	8.12%	8.35%	2.44%
**Obesity**	31.80%	29.25%	28.72%	26.68%	28.54%
**Depression**	6.80%	6.76%	7.09%	6.73%	1.94%
**Weight Loss (Abnormal Weight Gain)**	13.13%	11.62%	15.54%	10.09%	6.21%

**Table 3:** Genetic Regressions

Analysis	Odds Ratio (OR)/Beta	95% Confidence Interval (CI)	P-Value
**ADHD Diagnosis**	1.19	[1.10, 1.29]	<.001
**Stimulant Medication Use**	1.14	[1.07, 1.21]	<.001
**Stimulant Medication Use Without ADHD**	1.09	[1.01, 1.18]	0.024
**Stimulant Dosage**	−0.22	[−0.40, −0.05]	0.014
**Stimulant Dosage with ADHD**	−0.22	[−0.004, 0.44]	0.054

## Data Availability

Access to the Penn Medicine Biobank is available through https://pmbb.med.upenn.edu.

## Abbreviations

ADHD: Attention-deficit/hyperactivity disorder
PMBB: Penn Medicine BioBank
ICD: International Classification of Diseases
PGS: Polygenic scores
GWAS: Genome-wide association studies
EHR: Electronic health record
GSA V2: Illumina Global Screening Array
RGC: Regeneron Genetics Center
EUR: European
PCs: Principal components
